# Impact of Blood Transfusions After Kidney Transplantation on Clinical Outcomes and Broadness of Sensitisation

**DOI:** 10.1111/tan.71068

**Published:** 2026-11-05

**Authors:** Stella L. Gerber, Matthias Diebold, Gideon Hönger, Caroline Wehmeier, Thomas Wolff, Edin Mujagic, Corinne Widmer, Andreas Buser, Stefan Schaub

**Affiliations:** ^1^ Clinic for Transplantation Immunology and Nephrology University Hospital Basel Basel Switzerland; ^2^ HLA‐Diagnostics and Immunogenetics, Department of Laboratory Medicine University Hospital Basel Basel Switzerland; ^3^ Department of Vascular and Renal Transplant Surgery University Hospital Basel Basel Switzerland; ^4^ Department of Hematology and Laboratory Medicine University Hospital Basel Basel Switzerland; ^5^ Regional Blood Transfusion Service Swiss Red Cross, Northwestern Switzerland Basel Switzerland

**Keywords:** allograft rejection, erythrocyte transfusion, HLA sensitisation, kidney transplantation

## Abstract

Whether erythrocyte transfusion (ET) after kidney transplantation triggers formation of de novo donor‐specific HLA‐antibodies (HLA‐DSA) and rejection remains debated. If this were true, ET should broaden HLA sensitisation, reflected by increased calculated panel reactive antibodies (cPRA) and de novo HLA‐antibody specificities beyond HLA‐DSA. This has not been investigated in detail yet. In this retrospective study we included 519 kidney transplant recipients having single antigen bead analyses pretransplant and at one‐year posttransplant. ET was assessed both as a binary variable (with/without ET) and in quantity‐based groups (without ET, 1–2 ET, 3–5 ET, > 5 ET). No significant differences were found between patients with/without ET in the one‐year incidence of clinical rejection (19.7% vs. 15.3%, *p* = 0.43), clinical/subclinical rejection (30.0% vs. 28.6%, *p* = 0.86), TCMR (15.6% vs. 19.4%; *p* = 0.30) and AMR (15.6% vs. 10.1%; *p* = 0.19). Quantity‐based analysis also showed no significant differences, except for higher AMR incidence in patients receiving > 5 ET (*p* = 0.03). This association lost significance after multivariable adjustment in a time‐dependent Cox model (HR: 0.91 [95%‐CI: 0.52–1.59]; *p* = 0.74). De novo HLA‐DSA incidence was similar in patients with/without ET (3.5% vs. 3.1%, *p* = 0.80) and across quantity‐based groups (*p* = 0.17). cPRA change did not differ between patients with/without ET or across quantity‐based groups. No preferential sensitisation against highly prevalent HLA‐antigens Class I was observed (e.g., HLA‐A1 [0.4%], HLA‐A2 [0.8%], HLA‐B51 [0.8%] and HLA‐Cw7 [1.3%]). In conclusion, ET after kidney transplantation under tacrolimus/mycophenolate/prednisone‐based immunosuppression was not associated with rejection, de novo HLA‐DSA formation, and broadened HLA sensitisation arguing against the clinical need for HLA‐matched/selected ET in this setting.

**Abbreviations**
AMR: antibody‐mediated rejection
ATG: anti‐thymocyte globulin
cPRA: calculated panel reactive antibody
CXCL10: C‐X‐C motif chemokine ligand 10
DSA: donor‐specific antibody
ET: erythrocyte transfusion
MFI: mean fluorescence intensity
SAB: single antigen bead
TCMR: T cell‐mediated rejection

## 1 Introduction

Anaemia following kidney transplantation is highly prevalent, affecting 70%–80% of recipients in the first month and 30%–40% beyond 12 months. The causes are multifactorial, including perioperative blood loss, delayed graft function and the myelosuppressive effects of immunosuppressive agents [1]. Correspondingly, posttransplant erythrocyte transfusion (ET) is frequently required with reported transfusion rates ranging from 18% to 72% of patients within the first year [2].

Despite leucocyte depletion, ET retain immunogenic potential through residual lymphocytes [3] and HLA Class I antigens on the erythrocyte surface, which accumulate to a significant load per ET [4]. Indeed, in non‐immunosuppressed patients, leucocyte‐depleted ET are a well‐established cause of de novo HLA‐antibody formation, broadening the recipient's HLA sensitisation profile [5, 6, 7, 8, 9, 10, 11, 12]. This sensitising effect has been shown to be quantity‐dependent, increasing with the number of ET given [8].

Whether ET al. so triggers HLA‐antibody formation in the context of posttransplant immunosuppression remains a highly debated topic. The proposed mechanism suggests that HLA‐antigens present in the ET may stimulate antibody responses that cross‐react with donor HLA on the kidney allograft, thereby contributing to the formation of de novo donor‐specific HLA‐antibodies (HLA‐DSA) and subsequent allograft rejection, in particular antibody‐mediated rejection (AMR). The evidence for this is conflicting. While several studies have found an association between posttransplant ET and these events [13, 14, 15, 16, 17, 18], others have not [19, 20, 21, 22, 23, 24, 25, 26]. This variability across studies is likely related to heterogeneity in study design, population characteristics, assays used for HLA‐antibody detection and differences in immunosuppressive intensity.

If posttransplant ET were indeed the primary driver of de novo HLA‐DSA formation, one would expect sensitisation beyond HLA‐DSA, that is, against other HLA‐antigens present in the ET. This would induce a rise in calculated panel reactive antibodies (cPRA) and the detection of HLA‐antibodies against highly prevalent HLA‐antigens in the ET. To our knowledge, these outcomes have not yet been assessed in detail.

The aim of this study was to investigate the impact of ET as a binary variable (with/without ET) as well as in a quantity‐based manner in patients having a contemporary maintenance immunosuppression with tacrolimus/mycophenolate/prednisone on four outcomes: (i) occurrence of rejection, (ii) incidence of de novo HLA‐DSA, (iii) changes in cPRA from pretransplant to one‐year posttransplant and (iv) detection of new HLA‐antibody specificities one‐year posttransplant.

## 2 Materials and Methods

### 2.1 Study Population

This single‐centre, retrospective cohort study was approved by the local ethics committee of Northwestern and Central Switzerland (EKNZ 2026 – 00962). Between January 2017 and December 2024, a total of 630 kidney transplantations were performed at our institution. About 111 of 630 patients (17.6%) were excluded. The final study population consisted of 519 kidney transplant recipients with complete one‐year follow‐up, single‐antigen bead (SAB) analyses pretransplant and at one‐year posttransplant, as well as 11 loci high resolution HLA typing (Figure 1). Clinical, demographic and transplant‐related data were extracted retrospectively from the institutional electronic health record system and the transplant unit database at the University Hospital of Basel. Blood transfusion data were retrieved from the regional blood bank (www.blutspende‐nordwestschweiz.ch) by linking the patient's hospital ID, which ensured complete transfusion records. All ET were leucocyte‐depleted, namely < 1 × 10^6^ leucocytes per bag (national regulation since 2008).

**FIGURE 1:**
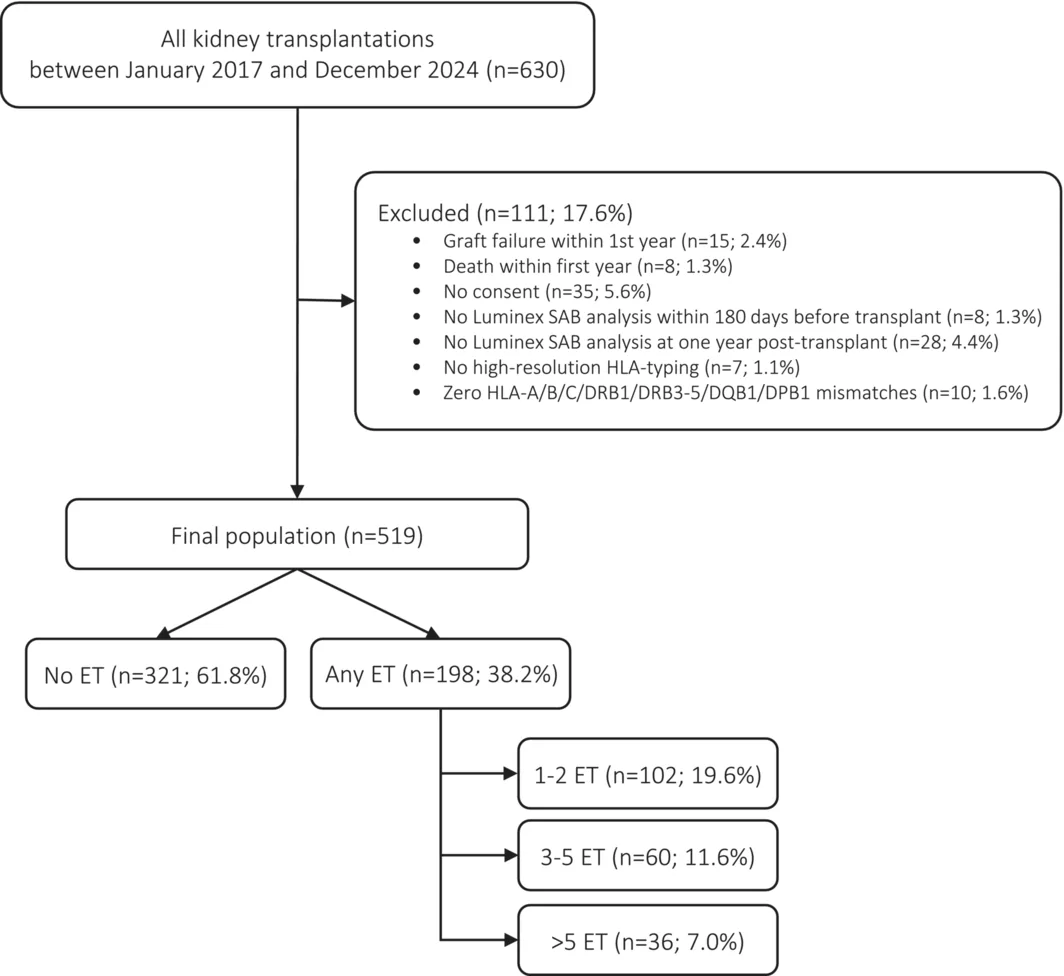
Patient flowchart. ET, erythrocyte transfusion; SAB, single antigen bead.

### 2.2 Exposure to Erythrocyte Transfusions

The cumulative number of ET received within the first‐year posttransplant was determined for each patient. The exposure to ET was first assessed as a binary variable (i.e., with/without ET). Subsequently, patients were further stratified by the quantity of ET into four arbitrary groups ensuring sufficient patients in each group for statistical analysis. The groups were defined as follows: no ET (*n* = 321), 1–2 ET (*n* = 102), 3–5 ET (*n* = 60), > 5 ET (*n* = 36) (Figure 1). Only 9/519 patients (1.7%) also received platelet transfusions (median 1; range: 1–7), and six of them were in the > 5 ET group.

### 2.3 HLA Antibody Analysis and Calculated Panel Reactive Antibodies

All HLA‐antibody analyses were performed using LABScreen SAB assays (www.thermofisher.com) on the Luminex platform. cPRA were determined using an in‐house calculator based on serological HLA equivalents observed in our own, predominantly Caucasian Swiss population. The cPRA values are in very good agreement with the French cPRA calculator (https://www.sfhitools.fr/cPRAdictor). The raw, baseline‐corrected MFI values of the SAB assays were used for cPRA calculation to avoid interpretation bias. If an HLA‐antigen included several alleles in the SAB panel, the antigen was only included for the cPRA calculation when the positive bead(s) represented > 90% of the allele frequency for the given HLA‐antigen. For example, if among the three A2‐beads only the A*02:01 bead was positive (population frequency 95% in a Caucasian population) then the A2 was included in the cPRA calculation. By contrast, if among the three A2‐beads only the A*02:03 bead was positive (population frequency < 1% in a Caucasian population) then the A2 was not included in the cPRA calculation. This approach was selected to prevent overestimation of cPRA due to allele‐specific antibodies against less frequent alleles.

### 2.4 Immunological Risk Stratification and Immunosuppression

The immunological risk stratification was based on the virtual crossmatch and has been described previously [27, 28]. Briefly, patients with HLA‐DSA determined by the virtual crossmatch were considered immunological high‐risk and received an induction therapy with anti‐thymocyte globulin (ATG; Grafalon or Thymoglobulin). Patients without HLA‐DSA were considered standard‐risk and received induction therapy with basiliximab. Initial maintenance immunosuppression consisted of tacrolimus, mycophenolate and prednisone. In high‐risk patients, this triple immunosuppression was maintained, whereas in standard‐risk patients, steroids were withdrawn beyond the third month if no rejection had occurred. The tacrolimus target trough levels were 8–10 μg/L until the third month, then 6–8 μg/L until the end of the first‐year posttransplant for both risk groups.

### 2.5 Outcomes of Interest

We defined four outcomes of interest. The first one was the occurrence of rejection classified according to the Banff 2022 criteria. Clinical biopsies were mainly performed in case of allograft dysfunction or delayed graft function persisting beyond Day 5. From January 2017 to September 2017 (*n* = 46) surveillance biopsies were routinely performed at 3‐ and 6‐month posttransplant. Thereafter, a urinary CXCL10‐based screening was used to guide surveillance biopsies at these time points, and a one‐year surveillance biopsy was performed as reported previously [29].

The second outcome of interest was the occurrence of de novo HLA‐DSA at one‐year posttransplant. All patients were screened for de novo HLA‐DSA at one‐year posttransplant. Additional analyses were performed if an allograft biopsy revealed an AMR phenotype. SAB with a mean fluorescence intensity (MFI) of > 500 was considered positive. Donor‐specificity was ensured by the virtual crossmatch approach relying on 11 loci high‐resolution HLA typing information.

The third outcome of interest was the evolution of cPRA from pretransplant to one‐year posttransplant. First, cPRA were determined for both time points applying three different MFI cutoffs (i.e., 500, 1000 and 3000). Then, the change of cPRA was calculated for the three different MFI cutoffs as cPRA_one‐year posttransplant_ − cPRA_pretransplant_. These ΔcPRA values were categorised into four groups: decreased cPRA, unchanged cPRA, increased cPRA ≤ 10% and increased cPRA > 10%. This arbitrary categorisation was implemented based on the assumption that a cPRA increase < 10% likely does not reflect a significant broadening of sensitisation.

The fourth outcome of interest was the detection of new HLA‐antibody specificities at one‐year posttransplant. We applied again three different MFI cutoffs (i.e., 500, 1000 and 3000) for SAB data obtained pretransplant and one‐year posttransplant. To illustrate this analysis, we give an example. If an HLA‐antibody against A*02:01 had a pretransplant MFI of 750 and a one‐year posttransplant MFI of 2000, it was considered as a new specificity applying the 1000 MFI cutoff. By contrast, it was not considered a new specificity using the 500 MFI cutoff (both values are > 500) or the 3000 MFI cutoff (both values < 3000). These new HLA‐antibody specificities were counted for all 519 transplantations and reported as a detection frequency (n/519). Next, these new HLA‐antibody were compared to a list of SAB, which we consider as ‘problematic’ due to a reaction frequency > 4% in unsensitised males across eight lots of SAB [30, 31] (Supplementary Table 1).

### 2.6 Statistical Analysis

Continuous variables are presented as mean ± standard deviation (SD) or median with interquartile range (IQR), depending on the distribution of the data assessed by the Shapiro–Wilk test. Categorical variables are presented as absolute numbers and percentages. For continuous variables, the *t*‐test or one‐way analysis of variance (ANOVA) test were used for normally distributed data, and Wilcoxon rank–sum test or Kruskal–Wallis test for non‐normally distributed data. For categorical variables, the Pearson chi‐squared test or Fisher's exact test was used as appropriate. The association between the quantity of ET and allograft rejection was assessed using time‐to‐event analysis (Kaplan–Meier method) and groups compared by the log‐rank test. To avoid immortal time bias, a time‐dependent Cox regression analysis was performed for the association between total number of ET as a continuous variable and clinical/subclinical rejection and clinical/subclinical AMR. Covariates were selected based on statistically significant differences between ET groups or established clinical relevance (i.e., recipient sex, kidney donor type [deceased vs. living donor], pretransplant HLA‐DSA, delayed graft function and HLA mismatch count). Due to collinearity with pretransplant HLA‐DSA, induction therapy and prior kidney transplant were not included in the model. The assumption of linearity was assessed using restricted cubic splines. As no evidence of non‐linearity was found, the linear model is reported, whereas the spline model is presented for graphical illustration. A two‐sided *p* value of < 0.05 was considered statistically significant. We used JMP 19.1.2 (SAS Institute, Cary, NC) and R‐4.4.1 (www.r‐project.org) for statistical analysis.

## 3 Results

### 3.1 Frequency of Blood Transfusion After Kidney Transplantation

About 198 of 519 patients (38.2%) received at least one ET within the first‐year posttransplant. Overall, 738 ET were given to these patients. The median haemoglobin at the time of ET was 69 g/L (IQR: 66–75). Most patients were transfused within the first week posttransplant (160/198; 80.8%) and the first month posttransplant (*n* = 185/198; 93.4%), respectively (Figure 2). Patients in the group with > 5 ET received a median of 8 ET (IQR: 6–12; range: 6–28).

**FIGURE 2:**
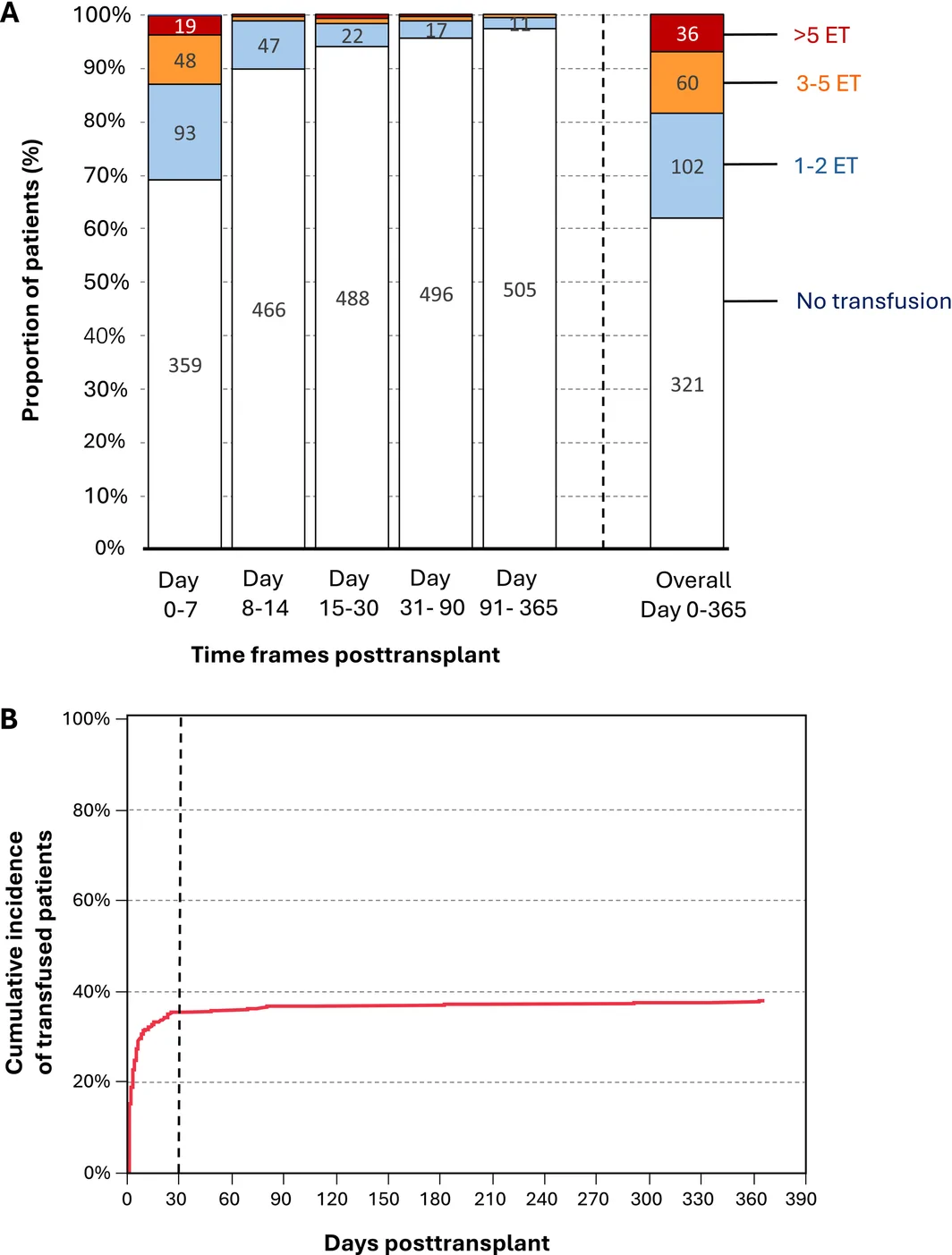
Erythrocyte transfusion (ET) frequency. (A) Proportion of patients receiving ET during defined time intervals and cumulatively over the first‐year posttransplant, grouped by the quantity of ET. Numbers within bars represent absolute patient counts. (B) Cumulative incidence of patients with ET in the first‐year posttransplant.

### 3.2 Baseline Characteristics

Demographic and clinical variables characterising the study population are summarised in Table 1. Patients with ET were significantly older (*p* = 0.0006), were more often female (*p* = 0.002), had received more often a deceased donor kidney (*p* < 0.0001), had more pretransplant sensitising events and higher cPRA, had more often pretransplant HLA‐DSA (*p* < 0.0001), received more often ATG‐induction (*p* < 0.0001), and experienced more often delayed graft function posttransplant (*p* < 0.0001) than patients without ET. The same observation was made for many variables when comparing the four groups stratified by the quantity of ET, often with a stepwise increase from 1–2 ET to 3–5 ET and > 5 ET (e.g., pretransplant HLA‐DSA, ATG‐induction, delayed graft function) (Table 1).

**TABLE 1:** Baseline characteristics by transfusion group.

Variable	Without ET (*n* = 321)	With ET (*n* = 198)	1–2 ET (*n* = 102)	3–5 ET (*n* = 60)	> 5 ET (*n* = 36)	*p‐*value^a^	*p‐*value^b^
Recipient age	53 (43–61)	58 (48–65)	59 (48–64)	56 (45–67)	60 (51–71)	0.0006	0.004
Female recipient, *n* (%)	87 (27.1%)	80 (40.4%)	42 (41.2%)	24 (40.0%)	14 (38.9%)	0.002	0.019
Cause of ESRD, *n* (%)						0.754	0.43
Vascular, hypertension	28 (8.7%)	23 (11.6%)	16 (15.7%)	2 (3.3%)	5 (13.9%)		
Polycystic kidney disease	71 (22.1%)	34 (17.2%)	18 (17.7%)	13 (21.7%)	3 (8.3%)		
Glomerulonephritis	114 (35.5%)	69 (34.9%)	29 (28.4%)	23 (38.3%)	17 (47.2%)		
Diabetes	22 (6.9%)	15 (7.6%)	7 (6.9%)	4 (6.7%)	4 (11.1%)		
Interstitial nephritis	16 (5.0%)	13 (6.6%)	7 (6.9%)	5 (8.3%)	1 (2.8%)		
Others	43 (13.4%)	29 (14.7%)	15 (14.7%)	10 (16.7%)	4 (11.1%)		
Unclear nephropathy	27 (8.4%)	15 (7.6%)	10 (9.8%)	3 (5.0%)	2 (5.6%)		
Renal replacement therapy modality, *n* (%)						0.029	0.111
Haemodialysis	211 (65.7%)	147 (74.2%)	72 (70.6%)	48 (80.0%)	27 (75.0%)		
Peritoneal dialysis	46 (14.3%)	29 (14.7%)	19 (18.6%)	5 (8.3%)	5 (13.9%)		
Pre‐emptive transplantation	64 (19.9%)	22 (11.1%)	11 (10.8%)	7 (11.7%)	4 (11.1%)		
Donor age	55 (45–62)	59 (50–65)	59 (51–64)	58 (43–66)	57 (50–65)	0.003	0.015
Deceased donor, *n* (%)	165 (51.4%)	148 (74.8%)	72 (70.6%)	47 (78.3%)	29 (80.6%)	< 0.0001	< 0.0001
Prior kidney transplant, *n* (%)	30 (9.4%)	42 (21.2%)	15 (14.7%)	15 (25.0%)	12 (33.3%)	0.0001	< 0.0001
Prior transfusions, *n* (%)	68 (21.2%)	63 (31.8%)	31 (30.4%)	19 (31.7%)	13 (36.1%)	0.007	0.05
Prior pregnancies, *n* (%)	56 (17.5%)	58 (29.3%)	30 (29.4%)	20 (33.3%)	8 (22.2%)	0.002	0.009
AB0i transplant, *n* (%)	16 (5.0%)	24 (12.1%)	16 (15.7%)	3 (5.0%)	5 (13.9%)	0.003	0.002
Pretransplant HLA‐DSA, *n* (%)	24 (7.5%)	40 (20.2%)	13 (12.8%)	16 (26.7%)	11 (30.6%)	< 0.0001	< 0.0001
HLA‐A/B/C/DRB1‐5/DQB1/DPB1 mismatch	8 (6–9)	8 (6–10)	8 (7–10)	8 (6–10)	8 (6–11)	0.036	0.14
Total eplet‐mismatch, mean (±SD)	59 (±1.2)	62 (±1.5)	62 (±2.1)	60 (±2.7)	63 (±3.5)	0.192	0.56
cPRA at transplantation, MFI 500	5.3 (0–35.6)	11.4 (0–63.1)	6.5 (0–47.1)	39.2 (1.1–81.2)	22.6 (0.1–63.4)	0.002	0.0003
cPRA at transplantation, MFI 1000	0 (0–20.5)	0.9 (0–46.1)	0 (0–29.1)	5.0 (0–79.8)	0.9 (0–54.1)	0.019	0.001
cPRA at transplantation, MFI 3000	0 (0–0)	0 (0–2.9)	0 (0–0)	0 (0–41.4)	0 (0–22.6)	0.037	0.007
cPRA Class I at transplantation, MFI 500	0 (0–13.0)	0.3 (0–36.1)	0 (0–26.3)	4.7 (0–43.4)	1.0 (0–45.6)	0.006	0.005
cPRA Class I at transplantation, MFI 1000	0 (0–1.4)	0 (0–9.2)	0 (0–5.1)	0.1 (0–24.7)	0 (0–33.5)	0.011	0.005
cPRA Class I at transplantation, MFI 3000	0 (0–0)	0 (0–0)	0 (0–0)	0 (0–1.1)	0 (0–20.3)	0.070	0.002
Induction, *n* (%)						< 0.0001	< 0.0001
ATG	35 (10.9%)	51 (25.8%)	17 (16.7%)	21 (35.0%)	13 (36.1%)		
Basiliximab	286 (89.1%)	147 (74.2%)	85 (83.3%)	39 (65.0%)	23 (63.9%)		
Maintenance immunosuppression, *n* (%)						1.000	0.23
Tacrolimus/Azathioprin/Prednisone	1 (0.3%)	1 (0.5%)	0 (0%)	0 (0%)	1 (2.8%)		
Tacrolimus/Mycophenolate/Prednisone	320 (99.7%)	197 (99.5%)	102 (100%)	60 (100%)	35 (97.2%)		
Haemoglobin pretransplant, mean (±SD)	117 (±0.8)	113 (±1.0)	113 (±1.5)	113 (±1.9)	112 (±2.5)	0.0005	0.007
Delayed graft function, *n* (%)	43 (13.4%)	80 (40.4%)	31 (30.4%)	29 (48.3%)	20 (55.6%)	< 0.0001	< 0.0001

Abbreviations: AB0i, AB0‐incompatible; ATG, anti‐thymocyte globulin; cPRA, calculated panel reactive antibodies; ESRD, end‐stage renal disease; ET, erythrocyte transfusion; HLA‐DSA, donor‐specific HLA‐antibodies; MFI, median fluorescence intensity.

^a^ *p* for no transfusion versus any transfusion: Chi^2^ or Fisher's exact test for categorical variables; Wilcoxon rank–sum or *t*‐test for continuous variables.

^b^ *p* across no transfusion, 1–2 ET, 3–5 ET, > 5 ET: Chi^2^ or Fisher's exact test for categorical variables; Kruskal–Wallis or one‐way ANOVA for continuous variables.

### 3.3 Development of Allograft Rejection

There was no statistically significant difference between patients with/without ET regarding the one‐year incidence of clinical rejection (19.7% vs. 15.3%; *p* = 0.43) and the one‐year incidence of clinical/subclinical rejection (30.0% vs. 28.3%; *p* = 0.86). Stratified by rejection phenotype, neither the incidence of clinical/subclinical T cell‐mediated rejection (TCMR) (15.6% vs. 19.4%; *p* = 0.30), nor the incidence of clinical/subclinical antibody‐mediated rejection (AMR) (15.6% vs. 10.1%; *p* = 0.19) showed statistically significant differences (Supplementary Figure 1). Next, we compared the four ET groups (i.e., > 5 ET, 3–5 ET, 1–2 ET, no ET) regarding the occurrence of rejection. The incidence of clinical rejection was not different among the four groups (*p* = 0.48). The incidence of clinical/subclinical rejection was numerically higher in patients with > 5 ET compared to the other groups (41.8% vs. 31.1% vs. 25.2% vs. 28.3%), but it did not reach statistical significance (*p* = 0.24). The incidence of clinical/subclinical TCMR was similar across all four groups (*p* = 0.65). The incidence of clinical/subclinical AMR differed significantly among the four groups, with the highest incidence in patients with > 5 ET (27.9% vs. 16.0% vs. 10.9% vs. 10.1%; *p* = 0.03) (Figure 3). In a time‐dependent Cox regression analysis there was no association between the total number of ET and the incidence of clinical/subclinical rejection (HR: 0.76 [95%‐CI: 0.52–1.11]; *p* = 0.15) or the incidence of clinical/subclinical AMR (HR: 0.91 [95%‐CI: 0.52–1.59]; *p* = 0.74) (Figure 4).

**FIGURE 3:**
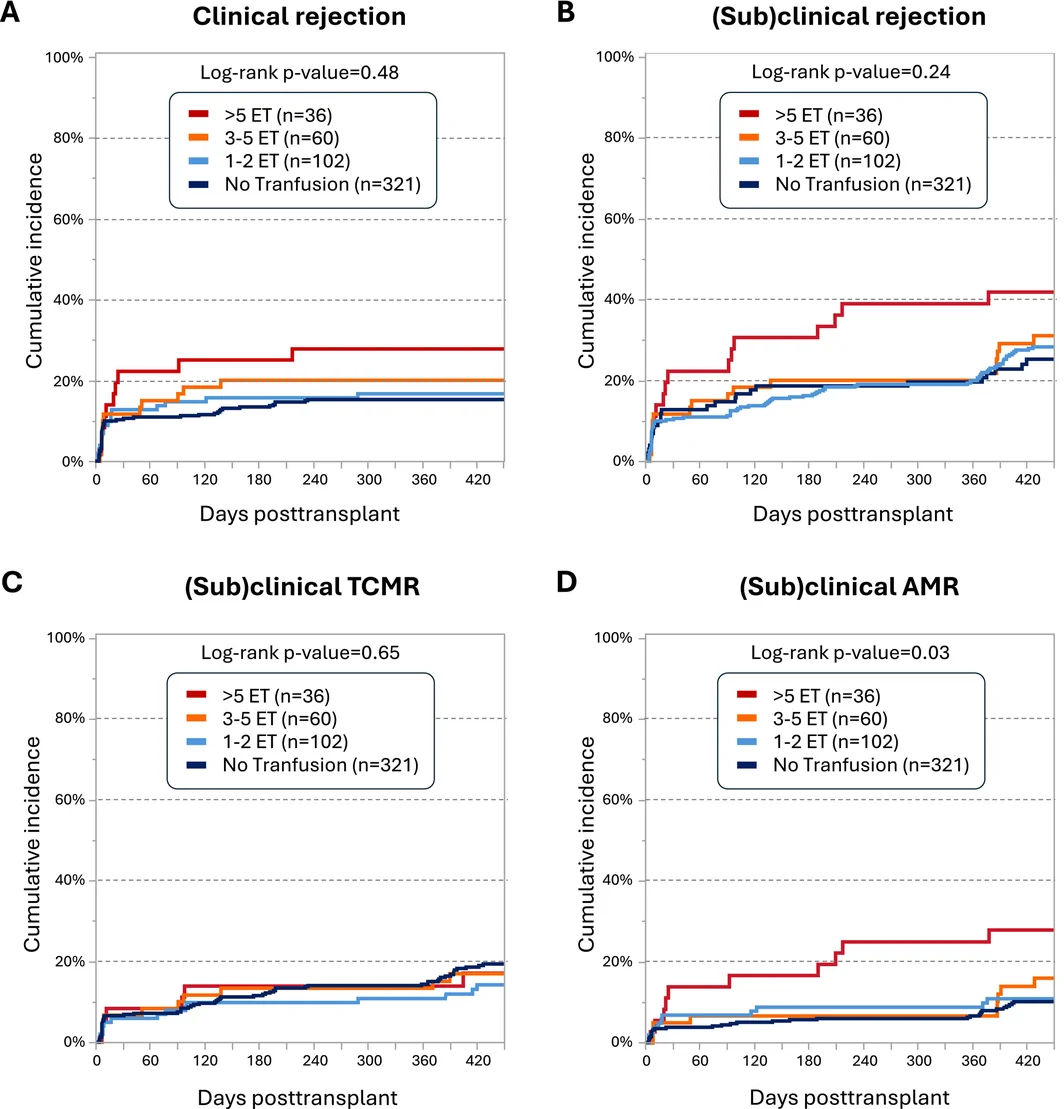
Incidence of rejection within the first‐year posttransplant, stratified by the quantity of erythrocyte transfusions (ET). (A) Cumulative incidence of clinical rejection. (B) Cumulative incidence of clinical or subclinical rejection. (C) Cumulative incidence of clinical or subclinical T cell‐mediated rejection (TCMR). (D) Cumulative incidence of clinical or subclinical antibody‐mediated rejection (AMR).

**FIGURE 4:**
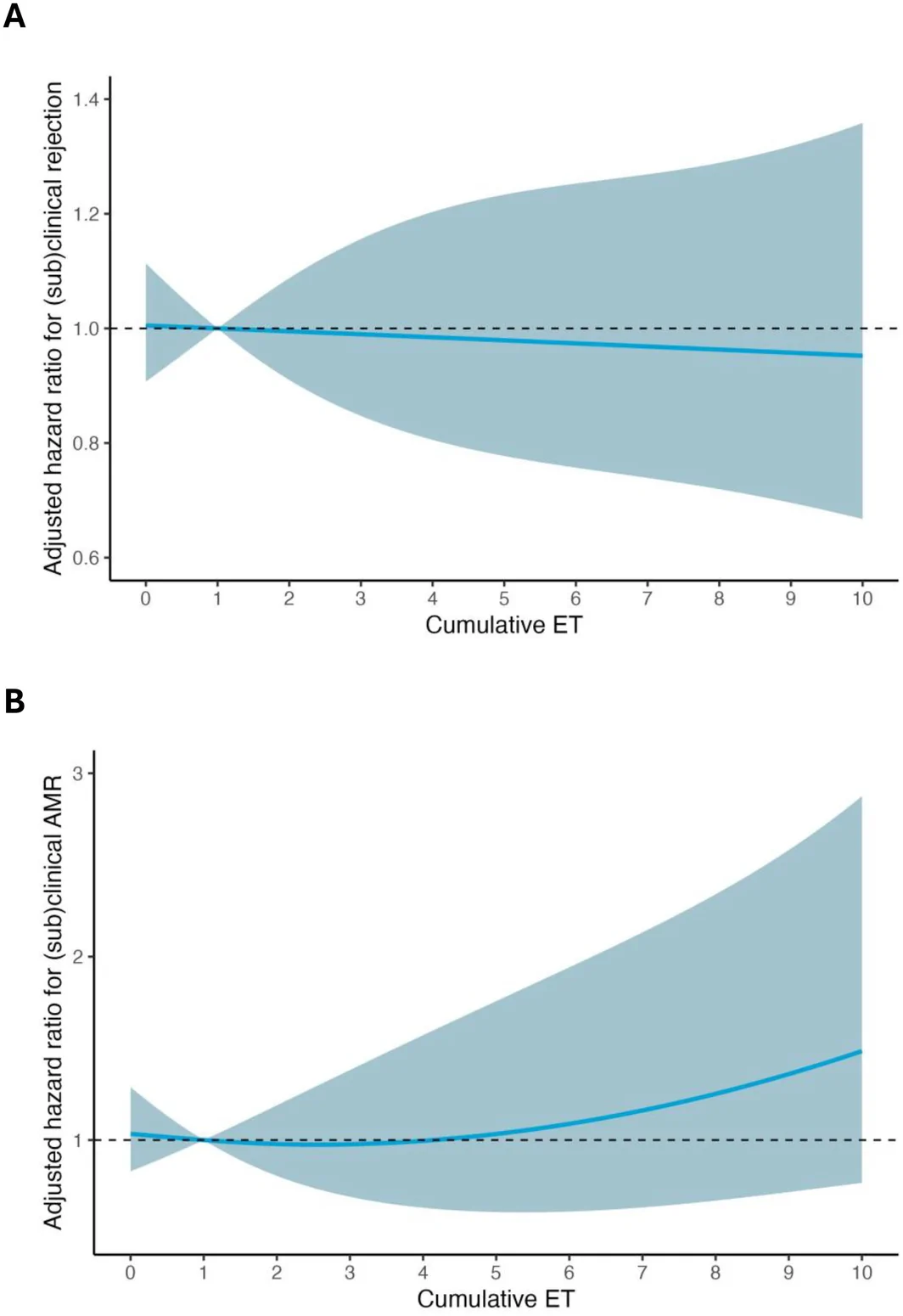
Association between cumulative erythrocyte transfusions (ET) and rejection. The solid line represents adjusted hazard ratios estimated from a time‐dependent Cox regression model using restricted cubic splines, with 1 ET as the reference. Shaded areas indicate 95% confidence intervals. Plots were truncated at 10 ET, because only 11 patients received > 10 ET. (A) Clinical or subclinical rejection. (B) Clinical or subclinical AMR.

### 3.4 Incidence of de Novo HLA‐DSA

De novo HLA‐DSA were detected in 17 out of 519 patients (3.3%) at one‐year posttransplant. Among these, HLA‐DSA Class II were the most frequent (*n* = 10), followed by Class I (*n* = 4) and combined Class I and II (*n* = 3). The incidence of de novo HLA‐DSA did not differ significantly between patients with/without ET within the first‐year posttransplant (3.5% vs. 3.1%; Fisher's exact test: *p* = 0.80). When stratified by quantity of ET, de novo HLA‐DSA were detected in 3.1% patients without ET, in 2.0% of patients with 1–2 ET, in 1.7% of patients with 3–5 ET and in 11.1% of patients with > 5 ET. Although a possible trend towards higher de novo HLA‐DSA incidence was observed in the group receiving > 5 ET, this difference did not reach statistical significance (Cochran–Armitage trend‐test: *p* = 0.17).

### 3.5 Evolution of cPRA

Overall, including Class I and II HLA‐antibody specificities using the 500 MFI cutoff, 40% of patients had decreasing cPRA, 39% of patients had unchanged cPRA, 11% had increasing cPRA ≤ 10% and 10% had increasing cPRA > 10%. Using the more robust 3000 MFI cutoff, 3% had increasing cPRA > 10%. When only Class I HLA‐antibodies were considered, 6% of patients had increasing cPRA > 10% using the 500 MFI cutoff, 3% using the 1000 MFI cutoff and 1% using the 3000 MFI cutoff. We observed no differences in cPRA changes between patients with/without ET for any MFI cutoff including only Class I HLA‐antibodies or Class I and II HLA‐antibodies (all comparisons with *p* ≥ 0.07; Supplementary Figure 2). The same results were obtained, if the four quantity‐based ET groups (i.e., no ET, 1–2 ET, 3–5 ET, > 5 ET) were compared, except for the 1000 MFI cutoff including only Class I HLA‐antibodies (*p* = 0.03) (Figure 5).

**FIGURE 5:**
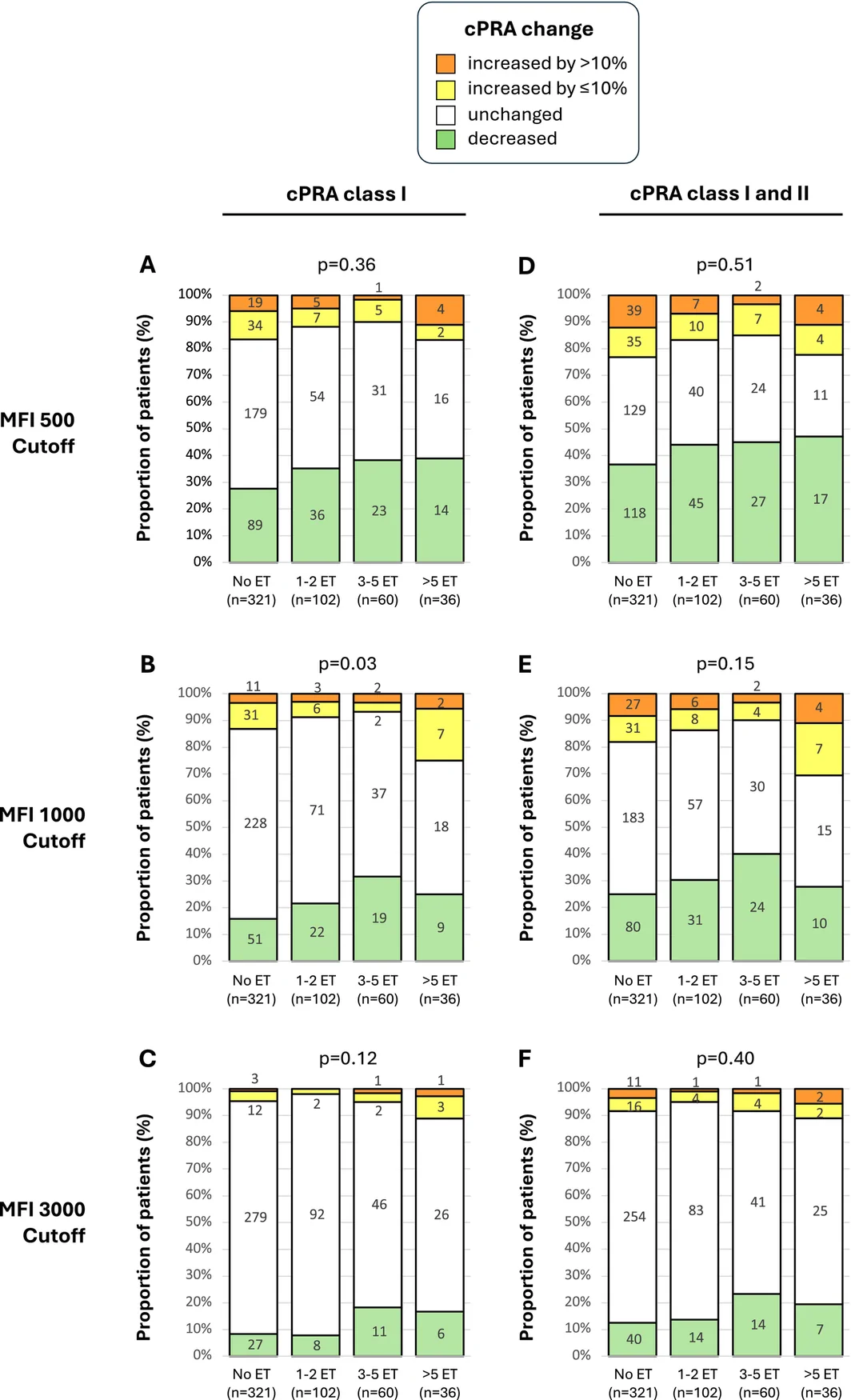
Distribution of cPRA changes stratified by the quantity of erythrocyte transfusions (ET) at different MFI cutoff. (A–C) HLA Class I at MFI 500, 1000 and 3000, respectively. (D–F) HLA Class I and II at MFI 500, 1000 and 3000, respectively. Numbers within bars represent absolute patient counts.

### 3.6 New HLA‐Antibody Specificities at One‐Year Posttransplant

Applying the 500 MFI cutoff, 106 new HLA‐antibody specificities were detected at one‐year posttransplant, 71 against HLA‐antigens Class I and 35 against HLA‐antigens Class II. At the 3000 MFI cutoff, 42 new HLA‐antibody specificities were detectable, 29 against HLA‐antigens Class I and 13 against HLA‐antigens Class II. The 10 most frequently detected new HLA‐antibody Class I and II specificities applying different MFI cutoffs are summarised in Table 2. Overall, the new specificities had low frequencies (maximum 4.4%) and were often directed against HLA‐antigens with a low population prevalence. In addition, a substantial proportion of the most frequently detected new HLA‐antibody specificities were directed against ‘problematic’ beads. Indeed, at MFI cutoffs of 500, 1000 and 3000, this applied to 14/20 (70%), 13/20 (65%) and 12/20 (60%) of specificities, respectively (Table 2).

**TABLE 2:** Top 10 most frequently detected new Class I and Class II HLA‐antibody specificities at one‐year posttransplant evaluated at MFI cutoffs of 500, 1000 and 3000, and sorted by their observed frequency.

HLA Class I (MFI 500 cutoff)			HLA Class I (MFI 1000 cutoff)			HLA Class I (MFI 3000 cutoff)		
Specificity	Pop. prev.	Freq.	Specificity	Pop. prev.	Freq.	Specificity	Pop. prev.	Freq.
Cw17 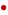	1.40%	4.40%	Cw17 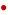	1.40%	2.10%	B45 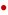	0.90%	0.60%
B37 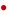	2.90%	2.50%	B37 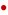	2.90%	1.90%	A23	4.70%	0.60%
B8 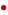	15.30%	2.10%	B8 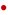	15.30%	1.20%	A32	8.40%	0.60%
B67	10.00%	1.90%	B73	0.30%	1.00%	B37 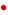	2.90%	0.40%
Cw4 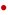	24.00%	1.70%	A80 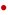	0.20%	1.00%	A24	21.20%	0.40%
B63	1.60%	1.50%	B45 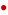	0.90%	1.00%	B67	10.00%	0.40%
B73	0.30%	1.50%	Cw4 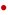	24.00%	0.80%	B82 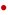	0.00%	0.40%
B76 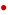	0.00%	1.50%	B63	1.60%	0.80%	B27	6.50%	0.40%
Cw7	50.10%	1.30%	B49	4.60%	0.80%	B38	4.60%	0.40%
A80 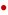	0.20%	1.30%	A2	46.10%	0.80%	Cw17	1.40%	0.20%

Bennett–Goodspeed antigens			Bennett–Goodspeed antigens			Bennett–Goodspeed antigens		
B7	20.30%	0.60%	B7	20.30%	0.00%	B7	20.30%	0.20%
B57	6.30%	0.80%	B57	6.30%	0.80%	B57	6.30%	0.20%
B58	2.80%	1.00%	B58	2.80%	0.20%	B58	2.80%	0.00%
A68	9.30%	0.40%	A68	9.30%	0.20%	A68	9.30%	0.40%
A69	0.40%	1.00%	A69	0.40%	0.40%	A69	0.40%	0.20%

HLA Class II (MFI 500 cutoff)			HLA Class II (MFI 1000 cutoff)			HLA Class II (MFI 3000 cutoff)		
Specificity	Pop. prev.	Freq.	Specificity	Pop. prev.	Freq.	Specificity	Pop. prev.	Freq.
DP14 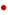	5.00%	3.50%	DR53 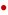	38.10%	1.70%	DP1 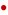	9.40%	1.20%
DR53 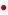	38.10%	3.10%	DQ8 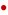	16.10%	1.50%	DP14 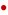	5.00%	0.40%
DR12	4.10%	3.10%	DP1 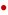	9.40%	1.50%	DR53 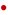	38.10%	0.40%
DR7 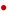	20.80%	2.90%	DR1 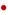	16.80%	1.30%	DR7 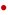	20.80%	0.40%
DQ8 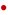	16.10%	2.10%	DR7 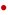	20.80%	1.20%	DR1 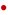	16.80%	0.40%
DR10	2.40%	1.50%	DR10	2.40%	1%	DQ7 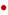	39.30%	0.40%
DR103 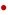	0.90%	1.50%	DR103	0.90%	1%	DQ8 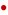	16.10%	0.20%
DP1 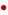	9.40%	1.30%	DR51 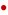	28.80%	1%	DR51 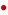	28.80%	0.20%
DR1 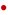	16.80%	1.30%	DP14 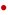	5.00%	0.80%	DR13	23.80%	0.20%
DR51 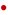	28.80%	1.00%	DR12	4.10%	0.80%	DQ2 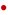	33.70%	0.20%

*Note:* For HLA Class I, the detection frequency of antibodies against Bennett–Goodspeed antigens (HLA‐B7, ‐B57/58 and ‐A68/69) at one‐year posttransplant is shown as well, given their particularly high expression on erythrocytes [32, 33]. 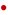 = Problematic bead, defined as a detection frequency over 4% in unsensitised males across eight lots of single antigen beads.

Abbreviations: Freq, frequency of detection; HLA, human leucocyte antigen; MFI, median fluorescence intensity; Pop Prev, prevalence of the antigen in the Swiss population.

No preferential HLA‐antibody formation was observed against high‐prevalence HLA‐antigens Class I (e.g., HLA‐A1 [0.4%], HLA‐A2 [0.8%] HLA‐A3 [0.2%], HLA‐B51 [0.8%], HLA‐Cw7 [1.3%]) or the Bennett–Goodspeed antigens (i.e., B7, B57, B58, A68 and A69), which are considered to have a high expression on erythrocytes [32, 33]. We give an example to illustrate the estimated versus the observed incidence of new HLA‐antibody formation. HLA‐A2 is present in approximately 45% of the Caucasian population and therefore also in 45% of random ET, which have the potential to induce an HLA‐A2 specific sensitisation in an HLA‐A2 negative patient. In our study population, 272/519 patients (52.4%) were HLA‐A2 negative. Among these 272 HLA‐A2 negative patients, 159 (58.5%) did not receive ET, and 113 (41.5%) were transfused with 1 to 28 ET. For each of these 113 patients, the probability of exposure to at least one HLA‐A2 positive ET was calculated. Assuming a sensitisation rate of 5% per ET, we would have expected 9/113 patients (8.0%) to develop an anti HLA‐A2 antibody (with a sensitisation rate of 10%, it would be 16/113 patients [14.2%]). However, only 1/113 patients (0.9%) had a detectable HLA‐A2 antibody at the 500 MFI cutoff, therefore lying far below the estimate. Notably, this patient fell into the 1–2 ET group (*n* = 59), whereas none of the patients in the 3–5 ET (*n* = 34) and > 5 ET (*n* = 20) groups developed a new HLA‐A2 antibody. Intriguingly, 2/159 HLA‐A2 negative patients (1.3%) who received no ET developed an HLA‐A2 antibody, which is not different from HLA‐A2 negative patients with ET (1/113 patients [0.9%]; *p* = 1.0).

### 3.7 Subgroup Analyses of Patients With Pretransplant HLA‐DSA

Sixty‐four patients had pretransplant HLA‐DSA. All but one patient had ATG induction, and 40/64 patients (63%) received ET: 1–2 ET (*n* = 13), 3–5 ET (*n* = 16), > 5 ET (*n* = 11). When patients were divided by transfusion status (i.e., with/without ET) or stratified by quantity of ET, we observed no difference in the incidence of clinical rejection (*p* = 0.28; *p* = 0.59), clinical/subclinical rejection (*p* = 0.90; *p* = 0.91), clinical/subclinical TCMR (*p* = 0.77; *p* = 0.44), clinical/subclinical AMR (*p* = 0.73; *p* = 0.75), development of de novo HLA‐DSA (*p* = 0.71; *p* = 0.51) or changes in cPRA (all *p* > 0.05). Furthermore, among 63 patients having received pre‐ and posttransplant ET, those with pretransplant HLA‐DSA (*n* = 23) did not have a higher incidence of rejection events than patients without pretransplant HLA‐DSA (*n* = 40) (all *p* ≥ 0.15). Specifically, the incidence of clinical/subclinical AMR was 9.1% in the HLA‐DSA group and 15.3% in the no HLA‐DSA group (*p* = 0.44).

## 4 Discussion

The key finding of this study is that neither the administration of ET per se nor the quantity of ET is associated with allograft rejection and development of de novo HLA‐DSA. The novelty in our analysis is the supporting evidence from a lack of change in cPRA and the absence of expected new HLA‐antibody specificities beyond HLA‐DSA. In addition, we used a robust statistical approach minimising immortal time bias in the multivariable regression analysis.

An important mechanistic question is whether ET can be a stronger immunological stimulus than the kidney allograft itself. This must be postulated, if ET is to be considered the driving force to induce de novo HLA‐DSA and rejection. Relevant determinants of an immune response in this setting are the duration of exposure, the load of HLA molecules, and the extent of immunosuppression. While the kidney allograft can permanently induce an immune response, the cells delivered by ET are only transiently present for a few weeks [34, 35].

Regarding the load of HLA molecules, we can provide the following estimation. Based on cell count and HLA molecule density data, a kidney allograft carries an estimated 10^16^ HLA Class I molecules on parenchymal cells [36, 37], and an estimated 10^14^ constitutively expressed HLA Class II molecules on microvascular endothelial cells [36, 38, 39, 40]. HLA Class II molecules are also expressed on interstitial dendritic cells and can be upregulated on tubular epithelial cells under inflammation. On the other hand, a standard ET delivers approximately 10^12^ erythrocytes, each carrying between 100 and 200 HLA Class I molecules on their surface [4], resulting in a total HLA Class I molecule load of 10^14^. According to leukodepletion standards, residual leucocytes must be below 10^6^ per ET, adding approximately 4 × 10^10^ HLA Class I and 2 × 10^10^ HLA Class II molecules [41]. Thus, the total HLA‐antigen load of a kidney allograft exceeds that of an ET approximately 100‐fold for HLA Class I molecules and approximately 10,000‐fold for HLA Class II molecules.

We agree with others that insufficient immunosuppression may help explain some discrepancies across published studies [21, 23]. For instance, Hassan et al. screened renal transplant recipients for de novo HLA‐DSA twice within the first week posttransplant, then at 1, 3, 6 and 12 months. They found that transfusion‐specific HLA‐antibodies (HLA‐TSA) developed against 61.5% of blood donors with nearly half of HLA‐TSA sharing specificity against the kidney allograft (i.e., HLA‐DSA). However, their maintenance immunosuppression mostly consisted of tacrolimus monotherapy after alemtuzumab induction, which is likely to result in a higher incidence of both HLA‐DSA and HLA‐TSA [16]. Interestingly, Fidler et al. found that exposure to ET posttransplant was associated with an AMR incidence of 65% in patients with pretransplant HLA‐DSA, who had also been transfused before transplantation (*n* = 20) suggesting an additive effect of ET in sensitised patients [18]. In contrast, we did not observe such an association among our 23 patients with pretransplant HLA‐DSA and ET pre‐ as well as posttransplant (AMR incidence of 9.1%). This difference could be explained by a much lower usage of ATG induction for patients with pretransplant HLA‐DSA in the Fidler study (5% vs. 98% in this study), a higher proportion of patients receiving cyclosporine as calcineurin‐inhibitor (24% vs. 0%), and different Banff versions for classification of AMR (2003 vs. 2022) [42].

A fundamental challenge in the assessment of ET on clinical outcomes is distinguishing causality from association. Only a prospective trial, randomising patients to receive ET or not, supported by a dense HLA‐antibody screening, could establish causality. However, it is unlikely that such a trial will be performed due to the ethical dilemma of withholding ET when clinically needed. Sophisticated statistical models can provide strong evidence for association but still cannot infer causality. Notably, one study applying a 7‐day time‐lag between ET and rejection found that the previously observed association disappeared [24].

This study has several strengths. To our knowledge, it is the first to assess systematically with high granularity whether ET under posttransplant immunosuppression leads to a broader HLA‐sensitisation (i.e., change of cPRA and emergence of new HLA‐antibody specificities beyond HLA‐DSA). Second, we applied three MFI cutoffs across all HLA‐antibody outcomes, which ensures robustness of the results obtained. Third, by modelling ET as a time‐dependent covariate in the Cox model we corrected for immortal time bias, a problem inherent to time‐to‐event analyses.

This study is not without limitations. First, it is a single centre study in a predominantly Caucasian population treated with tacrolimus/mycophenolate/prednisone‐based immunosuppression. Therefore, the results might not be applicable to other ethnicities or centres using different immunosuppression regimens. Second, SAB testing was performed at only two timepoints and therefore lacked in‐between measurements within the first‐year posttransplant. Consequently, it cannot be ruled out that transient new HLA‐antibody specificities induced by ET may have gone undetected, although most clinically relevant antibodies tend to persist [11]. Finally, the group of patients with > 5 ET was rather small (*n* = 36; 7% of the population) and hence, an effect of multiple ET on the investigated outcomes could have been missed due to lack of statistical power.

In conclusion, ET after kidney transplantation under tacrolimus/mycophenolate/prednisone‐ based immunosuppression was not associated with allograft rejection, de novo HLA‐DSA formation and broadening of HLA sensitisation. These findings argue against a clinical need for HLA matched/selected ET, at least in patients receiving a limited number of ET.

## Author Contributions

Participated in research design: S.L.G. and S.S. Participated in the writing of the paper: all. Participated in the performance of the research: all. Participated in data analysis: S.L.G., M.D. and S.S.

## Funding

The authors have nothing to report.

## Ethics Statement

This study was approved by the local ethics committee of North‐Western and Central Switzerland (EKNZ 2026 – 00962).

## Conflicts of Interest

The authors declare no conflicts of interest.

## Supporting information


**Supplementary Table 1** Problematic single‐antigen beads (SAB), defined as beads with an average detection frequency > 4% in unsensitised males across 8 lots of SAB panels.
**Supplementary Figure 1** Incidence of rejection within the first‐year posttransplant, comparing patients with/without erythrocyte transfusions (ET). (A) Cumulative incidence of clinical rejection. (B) Cumulative incidence of clinical or subclinical rejection. (C) Cumulative incidence of clinical or subclinical T cell‐mediated rejection (TCMR). (D) Cumulative incidence of clinical or subclinical antibody‐mediated rejection (AMR).
**Supplementary Figure 2** Distribution of cPRA‐changes comparing patients with/without erythrocyte transfusions (ET) at different MFI cutoff. (A–C) HLA Class I at MFI 500, 1000 and 3000, respectively. (D–F) HLA Class I and II at MFI 500, 1000 and 3000, respectively. Numbers within bars represent absolute patient counts.

## Data Availability

The data that support the findings of this study are available from the corresponding author upon reasonable request.
