# Supplementary material for: Impact of Blood Transfusions After Kidney Transplantation on Clinical Outcomes and Broadness of Sensitisation
Source: HLA. 2026 Nov 5;108(5):e71068. doi: 10.1111/tan.71068 (PMC13735889; doi:10.1111/tan.71068)

Supplementary Material

1) Supplementary Tables

Supplementary Table 1. Problematic single-antigen beads (SAB), defined as beads with an average detection frequency >4% in unsensitized males across 8 lots of SAB panels.

HLA class I		
**Antigen**	**Allele**	**Average detection frequency (%)**
B76	B*15:12	8.85
Cw17	C*17:01	7.45
A80	A*80:01	7.16
B37	B*37:01	6.39
B45	B*45:01	5.49
A34	A*34:01	5.01
Cw18	C*18:02	4.95
Cw9	C*03:03	4.93
Cw4	C*04:01	4.90
B46	B*46:01	4.83
A11	A*11:02	4.81
B82	B*82:01	4.79
B8	B*08:01	4.64
Cw12	C*12:03	4.64
A66	A*66:02	4.46
B44	B*44:02	4.39
Cw6	C*06:02	4.08

HLA class II		
**Antigen**	**Allele**	**Average detection frequency (%)**
**DP1**	DPB1*01:01 / DPA1*02:01	25.16
**DP5**	DPB1*05:01 / DPA1*02:02	13.49
**DR53**	DRB4*01:01	11.81
**DR53**	DRB4*01:03	8.16
**DP14**	DPB1*14:01 / DPA1*02:01	7.74
**DP1**	DPB1*01:01 / DPA1*01:03	6.91
**DQ7**	DQB1*03:01 / DQA1*05:03	6.88
**DR7**	DRB1*07:01	6.61
**DQ7**	DQB1*03:01 / DQA1*05:05	6.54
**DR4**	DRB1*04:04	6.49
**DP3**	DPB1*03:01 / DPA1*01:03	6.40
**DQ2**	DQB1*02:01 / DQA1*05:01	6.29

**DP6**	DPB1*06:01 / DPA1*01:03	6.14
**DQ8**	DQB1*03:02 / DQA1*03:01	6.03
**DQ8**	DQB1*03:02 / DQA1*03:02	5.75
**DQ7**	DQB1*03:01 / DQA1*02:01	5.30
**DR1**	DRB1*01:02	5.20
**DR51**	DRB5*01:01	5.16
**DQ9**	DQB1*03:03 / DQA1*03:02	4.74
**DQ9**	DQB1*03:03 / DQA1*03:01	4.69
**DQ7**	DQB1*03:01 / DQA1*03:01	4.68
**DR52**	DRB3*02:02	4.52
**DQ6**	DQB1*06:03 / DQA1*01:03	4.31
**DQ7**	DQB1*03:01 / DQA1*06:01	4.16
**DP11**	DPB1*11:01 / DPA1*02:02	4.16
**DP19**	DPB1*19:01 / DPA1*01:03	4.13
**DP13**	DPB1*13:01 / DPA1*03:01	4.09
**DR4**	DRB1*04:01	4.05

2) Supplementary Figure Legends

Supplementary Figure 1. Incidence of rejection within the first-year posttransplant, comparing patients with/without erythrocyte transfusions (ET). A: Cumulative incidence of clinical rejection. B: Cumulative incidence of clinical or subclinical rejection. C: Cumulative incidence of clinical or subclinical T cell-mediated rejection (TCMR). D: Cumulative incidence of clinical or subclinical antibody-mediated rejection (AMR).

Supplementary Figure 2. Distribution of cPRA-changes comparing patients with/without erythrocyte transfusions (ET) at different MFI cutoff. A-C: HLA class I at MFI 500, 1000 and 3000, respectively. D-F: HLA class I and II at MFI 500, 1000 and 3000, respectively. Numbers within bars represent absolute patient counts.

3) Supplementary Figures

Supplementary Figure 1.

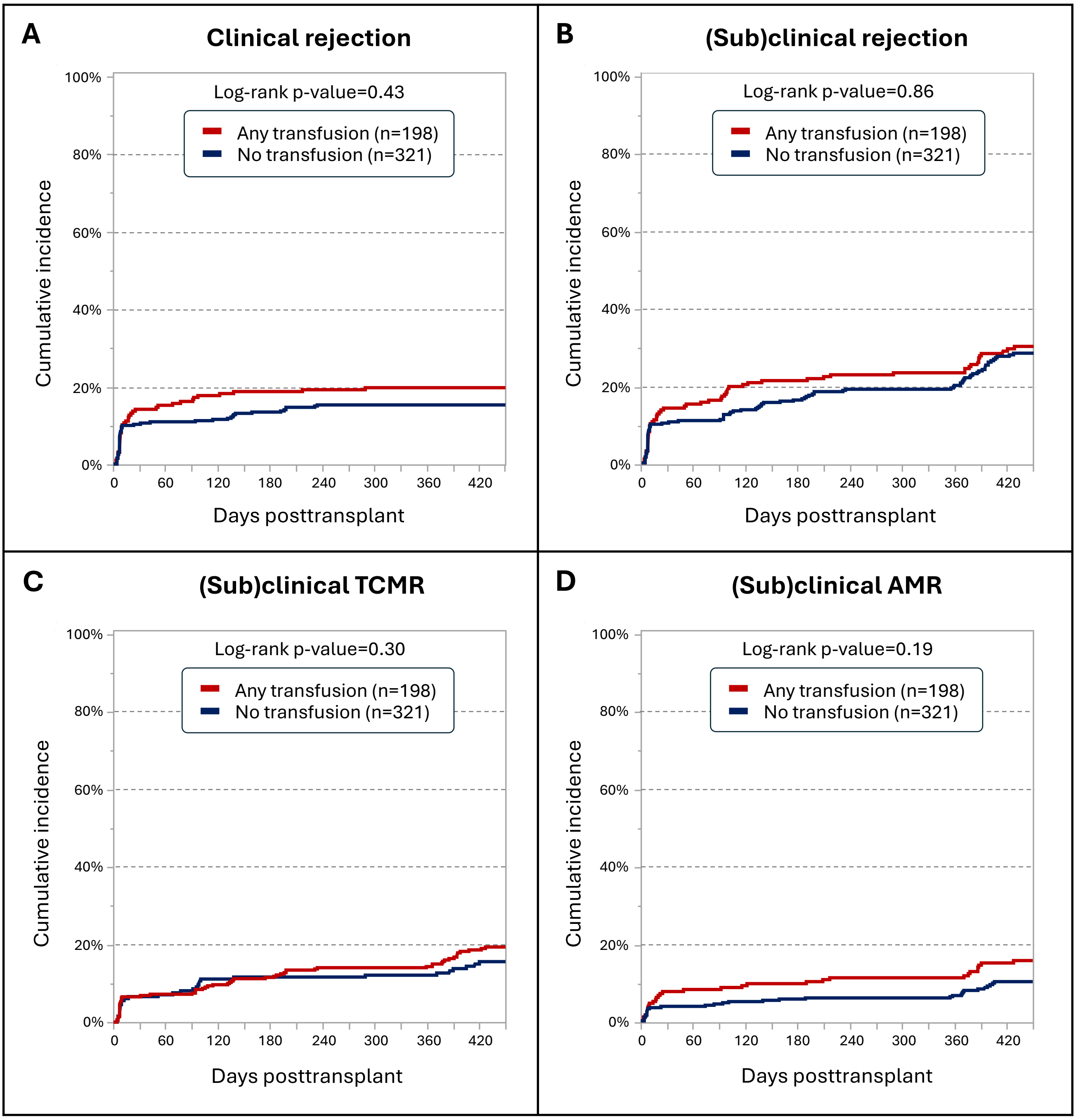

Supplementary figure 2.

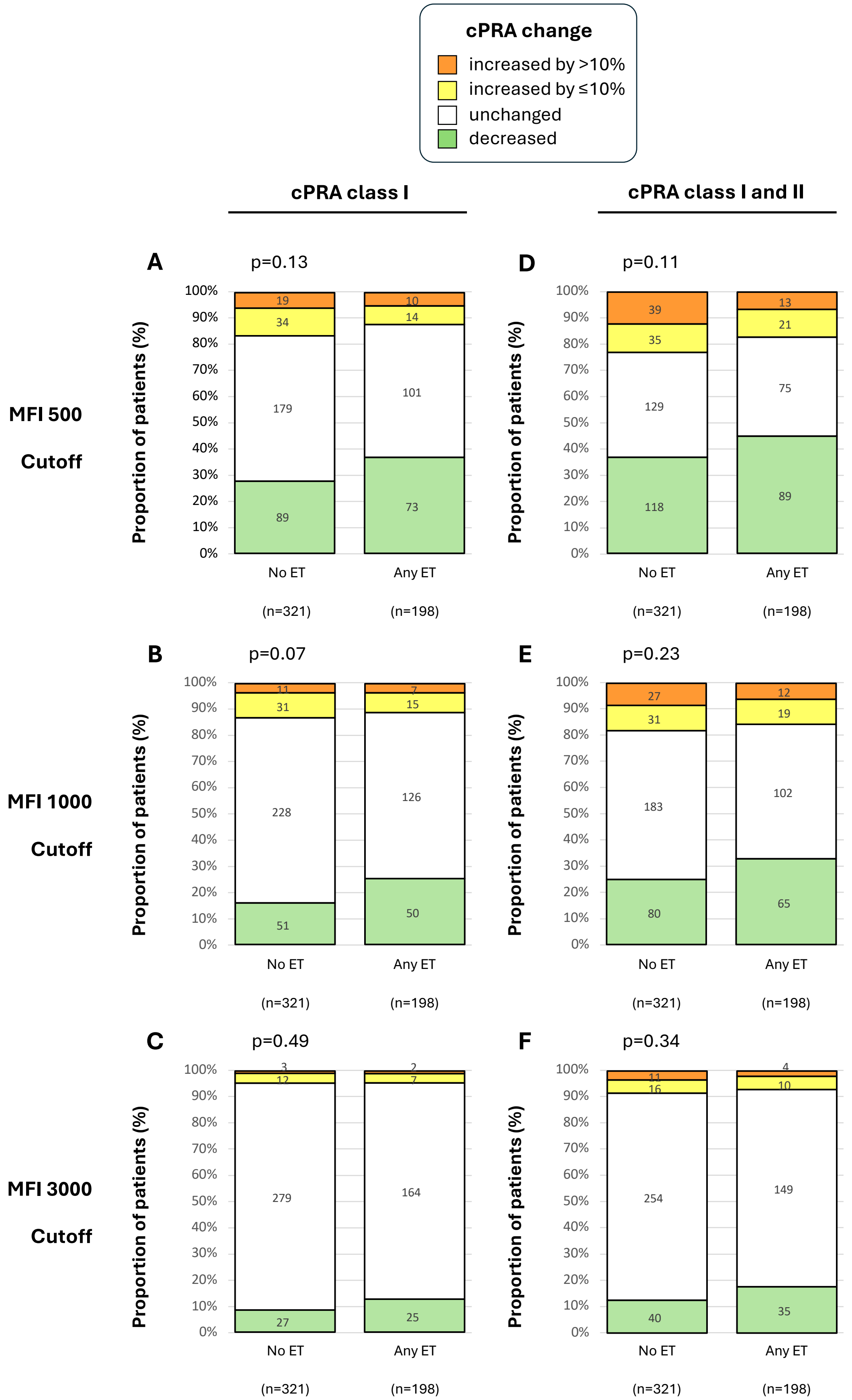

Supplement: Supplementary file 1 [file TAN-108-e71068-s001.pdf]
